# Supplementary material for: Effect of Inter-Reader Variability on Diffusion-Weighted MRI Apparent Diffusion Coefficient Measurements and Prediction of Pathologic Complete Response for Breast Cancer
Source: Tomography. 2022 Apr 22;8(3):1208–20. doi: 10.3390/tomography8030099 (PMC9149942; doi:10.3390/tomography8030099)
Supplement: Supplementary file 1 [file tomography-08-00099-s001.zip › tomography-1601717-supplementary.pdf]

Supplements

Supplemental Table S1. ICC values for ADC metrics

	ADC metrics	ICC (95% CI)		
		**Multiple-slice restricted ROI**	**Single-slice restricted ROI**	**Single-slice tumor ROI**
T0	MEAN	0.98 (0.97,0.987)	0.964 (0.947,0.975)	0.978 (0.963,0.986)
	MINIMUM	0.791 (0.655,0.868)	0.821 (0.738,0.877)	0.929 (0.895,0.953)
	MAXIMUM	0.808 (0.73,0.865)	0.83 (0.759,0.881)	0.88 (0.829,0.917)
	PCTL 05	0.942 (0.909,0.962)	0.939 (0.911,0.958)	0.965 (0.947,0.976)
	PCTL 15	0.975 (0.962,0.984)	0.963 (0.946,0.975)	0.975 (0.961,0.984)
	PCTL 25	0.968 (0.953,0.979)	0.954 (0.934,0.969)	0.977 (0.965,0.984)
	PCTL 50	0.981 (0.971,0.987)	0.964 (0.947,0.975)	0.974 (0.956,0.984)
	PCTL 75	0.973 (0.961,0.982)	0.946 (0.922,0.963)	0.964 (0.943,0.976)
	PCTL 95	0.93 (0.906,0.955)	0.92 (0.885,0.945)	0.936 (0.902,0.958)
T1	MEAN	0.983 (0.974,0.988)	0.98 (0.97,0.986)	0.963 (0.941,0.976)
	MINIMUM	0.94 (0.912,0.958)	0.95 (0.925,0.966)	0.952 (0.93,0.967)
	MAXIMUM	0.848 (0.785,0.894)	0.881 (0.831,0.918)	0.929 (0.893,0.952)
	PCTL 05	0.964 (0.948,0.975)	0.961 (0.943,0.973)	0.957 (0.937,0.97)
	PCTL 15	0.968 (0.953,0.978)	0.966 (0.951,0.977)	0.934 (0.905,0.955)
	PCTL 25	0.978 (0.967,0.985)	0.978 (0.967,0.985)	0.955 (0.934,0.969)
	PCTL 50	0.975 (0.96,0.984)	0.974 (0.962,0.982)	0.953 (0.925,0.969)
	PCTL 75	0.971 (0.957,0.98)	0.968 (0.954,0.978)	0.964 (0.945,0.976)
	PCTL 95	0.909 (0.869,0.937)	0.914 (0.876,0.941)	0.937 (0.906,0.957)

ΔADC%	MEAN	0.973 (0.961,0.982)	0.94 (0.912,0.959)	0.937 (0.908,0.957)
	MINIMUM	0.045 (-0.145,0.232)	0.083 (-0.109,0.27)	0.858 (0.797,0.901)
	MAXIMUM	0.71 (0.599,0.794)	0.723 (0.616,0.804)	0.781 (0.692,0.846)
	PCTL 05	0.909 (0.866,0.939)	0.921 (0.885,0.946)	0.931 (0.9,0.953)
	PCTL 15	0.951 (0.928,0.967)	0.942 (0.915,0.96)	0.885 (0.835,0.921)
	PCTL 25	0.955 (0.934,0.969)	0.944 (0.919,0.962)	0.921 (0.885,0.946)
	PCTL 50	0.961 (0.943,0.974)	0.936 (0.907,0.956)	0.931 (0.899,0.953)
	PCTL 75	0.954 (0.933,0.969)	0.899 (0.854,0.93)	0.917 (0.88,0.943)
	PCTL 95	0.844 (0.778,0.892)	0.784 (0.696,0.848)	0.85 (0.786,0.896)

Supplemental Table S2. AUC values for predicting pCR using ROIs delineated by two readers

**PERCENT CHANGE of ADC metrics**	**AUC (95% CI)**					
	**Multiple-slice restricted ROI**		**Single-slice restricted ROI**		**Single-slice tumor ROI**	
	**Reader 1**	**Reader 2**	**Reader 1**	**Reader 2**	**Reader 1**	**Reader 2**
MEAN	**0.653 (0.515,0.79)**	**0.668 (0.529,0.807)**	**0.638 (0.504,0.773)**	**0.631 (0.501,0.761)**	0.579 (0.45,0.708)	0.559 (0.429,0.689)
MINIMUM	0.619 (0.479,0.758)	**0.668 (0.534,0.803)**	0.599 (0.471,0.727)	0.591 (0.465,0.716)	0.602 (0.475,0.73)	0.597 (0.467,0.728)
MAXIMUM	0.489 (0.364,0.6ne14)	0.488 (0.367,0.608)	0.541 (0.417,0.664)	0.547 (0.424,0.669)	0.496 (0.374,0.618)	0.384 (0.266,0.503)
PCTL 05	0.591 (0.449,0.734)	0.637 (0.497,0.777)	0.589 (0.454,0.724)	0.599 (0.468,0.731)	0.528 (0.396,0.66)	0.538 (0.403,0.674)
PCTL 15	0.631 (0.493,0.769)	0.63 (0.488,0.772)	0.598 (0.464,0.732)	0.591 (0.457,0.725)	0.561 (0.431,0.692)	0.549 (0.418,0.68)
PCTL 25	0.633 (0.496,0.77)	**0.655 (0.514,0.797)**	0.611 (0.476,0.746)	0.608 (0.474,0.742)	0.556 (0.421,0.691)	0.578 (0.444,0.712)

PCTL 50	0.627 (0.492,0.762)	0.625 (0.486,0.763)	0.627 (0.493,0.761)	0.611 (0.481,0.742)	0.57 (0.439, 0.7)	0.547 (0.417,0.677)
PCTL 75	0.624 (0.491,0.758)	0.622 (0.487,0.758)	0.609 (0.476,0.742)	0.595 (0.466,0.723)	0.554 (0.431, 0.677)	0.542 (0.414,0.669)
PCTL 95	0.598 (0.471,0.726)	0.608 (0.479,0.737)	0.615 (0.486,0.743)	0.573 (0.447,0.7)	0.557 (0.434, 0.68)	0.48 (0.361,0.6)

Numbers in bold had 95% CI above 0.5
